# Utilizing standardized nursing terminologies in implementing an AI-powered fall-prevention tool to improve patient outcomes: a multihospital study

**DOI:** 10.1093/jamia/ocad145

**Published:** 2023-07-28

**Authors:** Insook Cho, Jiseon Cho, Jeong Hee Hong, Wha Suk Choe, HyeKyeong Shin

**Affiliations:** Nursing Department, Inha University, Incheon, Republic of Korea; Division of General Internal Medicine, The Center for Patient Safety Research and Practice, Brigham and Women’s Hospital, Boston, Massachusetts, USA; Department of Nursing, National Health Insurance Service Ilsan Hospital, Gyeonggi-do, Republic of Korea; Department of Nursing, Samsung Medical Center, Seoul, Republic of Korea; Department of Nursing, Inha University Hospital, Incheon, Republic of Korea; Graduate School, Nursing Department, Inha University, Incheon, Republic of Korea

**Keywords:** standardized nursing terminologies, AI-powered clinical decision support tool, fall prevention, semantic interoperability, patient outcomes

## Abstract

**Objectives:**

Standardized nursing terminologies (SNTs) are necessary to ensure consistent knowledge expression and compare the effectiveness of nursing practice across settings. This study investigated whether SNTs can support semantic interoperability and outcoming tracking over time by implementing an AI-powered CDS tool for fall prevention across multiple EMR systems.

**Materials and Methods:**

The study involved 3 tertiary academic hospitals and 1 public hospital with different EMR systems and nursing terms, and employed an AI-powered CDS tool that determines the fall risk within the next hour (prediction model) and recommends tailored care plans (CDS functions; represented by SNTs). The prediction model was mapped to local data elements and optimized using local data sets. The local nursing statements in CDS functions were mapped using an ICNP-based inpatient fall-prevention catalog. Four implementation models were compared, and patient outcomes and nursing activities were observed longitudinally at one site.

**Results:**

The postimplementation approach was practical for disseminating the AI-powered CDS tool for nursing. The 4 hospitals successfully implemented prediction models with little performance variation; the AUROCs were 0.8051–0.9581. The nursing process data contributed markedly to fall-risk predictions. The local nursing statements on preventing falls covered 48.0%–86.7% of statements. There was no significant longitudinal decrease in the fall rate (*P *=* *.160, 95% CI = −1.21 to 0.21 per 1000 hospital days), but rates of interventions provided by nurses were notably increased.

**Conclusion:**

SNTs contributed to achieving semantic interoperability among multiple EMR systems to disseminate AI-powered CDS tools and automatically track nursing and patient outcomes.

## INTRODUCTION

Healthcare computerization and the development of standardized nursing terminologies (SNTs) have led to electronic medical/health records (EMRs/EHRs) including nursing assessments. SNTs can represent nursing data in a formal, computable format in electronic clinical systems that is consistent with reference terminologies.^1^^,^^2^ Various clinical systems have adopted SNTs to aggregate and analyze nursing data for clinical, resource-management, and financial purposes. The International Organization for Standardization (ISO) approved the International Standard for the Representation of Nursing Diagnoses and Interventions, which assists in integrating each terminology into computer systems and ensures their interoperability.^1^

Nursing documentation data in EHRs include nursing services, patient demographics, progress notes, assessment data, and care plans.^3^ Nursing data specifically refer to care plans, and the problems, target outcomes, and planned and implemented interventions for their patients, and are rich sources of clinical reasoning.^4^ Nursing data are used for communication within healthcare teams via centralized, accessible patient-information repositories. They are also used by healthcare providers to make informed decisions on patient care and identify trends and patterns for informing treatment plans. Nursing data are also a critical component of EHRs for improving patient outcomes and ensuring the safe delivery of high-quality care.^5–7^

The Future of Nursing 2020–2030 report focused on nurse roles in transforming healthcare by addressing the challenges and opportunities they will face in the near future.^8^ That report recognized the potential of technology in transforming the delivery of nursing care and improving patient outcomes, and suggested that nurses can leverage various technologies such as EHRs, telehealth, big data, artificial intelligence (AI), and clinical decision support (CDS). Combining these technologies with AI-powered CDS tools can support tasks including screening and triage, diagnosis, treatment planning, prognosis, and treatment recommendations.^9^ These tools can improve the accuracy and efficiency of care and provide real-time support for nursing decisions alongside evidence-based recommendations.^10^ However, nurses have far less experience with AI-powered tools than with other tools that have been assessed in many trials and healthcare research studies.^11–13^ Nurses must therefore be introduced to and familiarized with tangible clinical examples of these tools and understand their relationship with SNTs. Nurses can make vital contributions to healthcare transformation and health equity by incorporating innovative technologies.^5^^,^^7^

This study introduced SNT usage for supporting the semantic interoperability of AI-powered-tool implementation across multiple EMR systems using a postimplementation approach and time-dependent pattern analysis. The tool was designed to inform nurses of patient-level fall risks using nursing documentation data from EMR systems and to recommend risk-targeted, tailored interventions. This study aimed to use a predictive concept model based on clinical guidelines for fall prevention to: (1) determine the feasibility of implementing locally optimized prediction models that were integrated with 4 distinct EMR systems using SNTs, (2) compare the differences among the nursing contents in local EMR systems based on standard statements for fall prevention, and (3) identify longitudinal patterns of patient outcomes and nursing activities for fall prevention.

## BACKGROUND

### Inpatient falls

Inpatient falls are a well-known preventable adverse event closely related to the quality of nursing care.^14^ This negative outcome can cause physical injury and have estimated healthcare costs of US$351–13 616 per patient in the United States.^15^^,^^16^ Heuristic tools with proven clinical performance include the Morse Fall Scale, St. Thomas’ Risk Assessment Tool in Falling Elderly Inpatients (STRATIFY), Hendrich II Fall Risk Model, and Johns Hopkins Fall Risk Assessment Tool. However, several questions have been raised about the clinical usefulness of these tools.^17^ We have found that some nurses employ simple copy-and-paste behaviors when using these tools due to their cognitive burden, making fall prevention in hospitals unsatisfactory.^18^

Several studies have developed various prediction models for inpatient falls through widespread EHR use and clinical big-data use, and advances in data analysis techniques.^19–22^ However, these studies had several limitations. First, their final models did not include the preventive efforts made by nurses. Second, they focused on comparing machine-learning algorithms rather than clinical applications, resulting in the selection of not-evidence-based risk factors. Third, most models were developed using single-site data and not validated among different sites or different EMR systems, thereby ignoring issues with SNTs.

Numerous studies have investigated inpatient falls as a practical problem with nursing quality, but outcome improvements have been slow.^23^ Better approaches are required, particularly given the increases in the number of hospitalized older patients and disease complexity.

### Development of an AI-powered CDS tool using SNTs

Our previously described AI-powered CDS tool for preventing inpatient falls was developed in 2 phases^24^^,^^25^: (1) developing a fall prediction model using retrospective data and (2) integrating the model with EMR systems and implementing it using CDS functions. The tool aimed to determine the fall risk of a patient within the next hour and the relevant risk factors, to guide a care plan and recommend tailored interventions.

#### Phase I: risk prediction model

The model needed to be accepted by nurses in their practical work and be deployed across healthcare organizations. Three important design principles were applied. First, we used both evidence-based and data-driven approaches, by considering previously discovered evidence and clinical guidelines in the design phase, and then applying nursing data from the EMR systems. Second, the machine-learning algorithm of the CDS tool must be understandable by and informative to nurses, meaning that it should be interpretable and explainable. Machine-learning interpretability reflects whether decision-makers (end users) understand the model, while explainability indicates what the model presents to decision-makers that results in them trusting its functionality.^26^ Third, the machine-learning algorithm should be shareable and comparable among the EMR systems of different hospitals to avoid reinventing the wheel. To satisfy these requirements, we adopted Bayesian networks to present nursing knowledge and graphs to present the formalism that transparently represented probabilistic relationships and conditional independence. Such a network provides a transparent mechanism to communicate what the system knows about the probabilistic relationships among the network nodes. A Bayesian network calculates the posterior probabilities of the conditions at runtime.^27^

**Figure 1.**
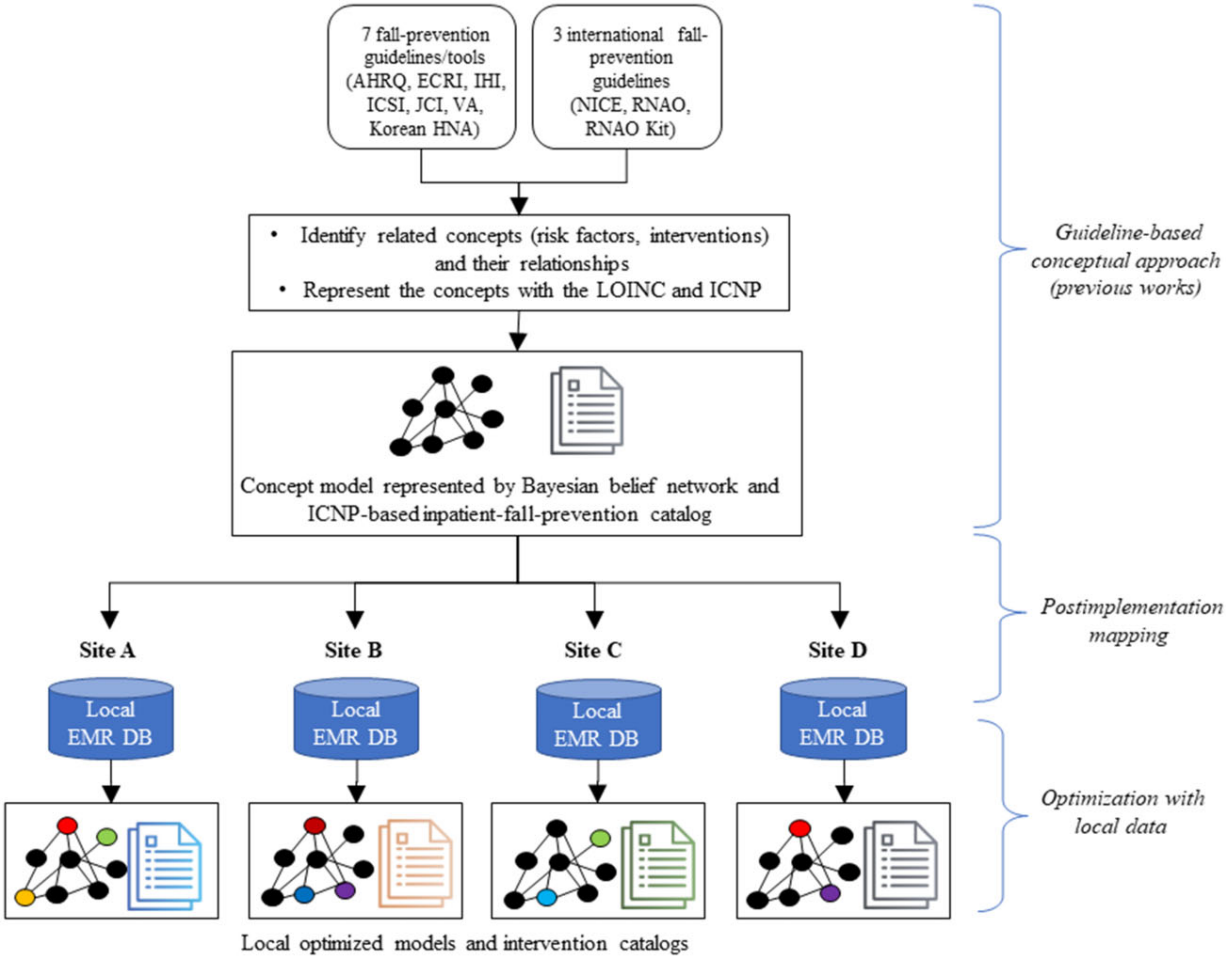
Post-implementation approach for the development of AI-powered clinical decision support tools for multihospitals, AHRQ: Agency for Healthcare Research and Quality; IHI: Institute for Healthcare Improvement; ICSI: Institute for Clinical Systems Improvement; JCI: Joint Commission Center for Transforming Healthcare; VA: Veterans Affairs, Veterans Affairs National Center for Patient Safety; Korean HNA: Korean Hospital Nurses Association; NICE: National Institute for Health and Care Excellence; RNAO: Registered Nurses’ Association of Ontario; LOINC: Logical Observation Identifiers Names and Codes; ICNP^®^: International Classification for Nursing Practice.

Figure 1 shows the postimplementation mapping approach that employed the Logical Observation Identifiers Names and Codes (LOINC) and International Classification for Nursing Practice (ICNP). The LOINC provides a method of systematically standardizing observations used during the nursing process, including assessments, goals, and outcomes, facilitates the exchange and pooling of results.^28^ The ICNP is a coded standard terminology developed by the International Council of Nurses (ICN) to represent nursing knowledge and care in EHRs, and is accepted by the World Health Organization (WHO) as an international classification.^2^ This formal mapping process was already defined in previous studies on eMeasurement populations from EHR systems for inpatient falls^29^ and in the ICNP-based inpatient fall-prevention catalog (‘The Catalog’) from practice guidelines.^25^

The risk factors and intervention recommendations for falls were identified from 7 clinical guidelines and toolkits.^30–35^ These assessments and interventions were represented using nursing concepts based on SNTs and were used to construct the Bayesian network with the relationships within it, which we called a concept model for predicting fall risks (Supplementary Table S1). The concept model was mapped using the local data elements of EMR systems (structured data and semistructured nursing statements), which were populated from a local nursing dictionary. A research team was placed at each site for the local mapping, comprising nursing informaticians from academia, nurse managers with PhDs, and experienced staff nurses. Two nursing informaticians worked with the local research teams to consider local nursing record systems and practice patterns in order to maintain the semantic interoperability of the process (Supplementary Figure S1). The performance of each implementation model was optimized through training and validation using local data sets.

#### Phase II: defining and implementing the CDS function

The 4 optimized implementation models were integrated into a CDS tool that would determine the individual fall risk within the next hour and the associated risk factors. This tool could also suggest care plans and tailored interventions based on those risk factors. To realize these functions, the CDS tool requires domain content that describes the relationship between the relevant clinical data and nursing-process components. The Catalog was used as a clinical content model for fall prevention, which was developed based on the conceptual framework of the International Classification for Patient Safety (ICPS) and 4 international guidelines.^36–39^ The care plans of The Catalog were based on the ICNP,^25^ consisting of 18 nursing care elements and 141 terms, with 98 terms among 14 care elements being used in this study; the care elements that corresponded to postfall huddle and patient outcomes were excluded. The intervention in The Catalog were grouped into 6 risk-factor categories: cognition, toileting, mobility, medications, sensory, and sleep. For example, if a patient is predicted to be at risk due to impaired cognition, the CDS tool will recommend providing hourly nursing rounds, caregivers, or bedside sitters, or installing alarm monitors such as video, motion detection, or sensor-based tools. If a patient is taking diuretic and sedative medications, the tailored recommendation will include ceasing diuretic medication use before 7pm, monitoring the serum potassium level, assessing dizziness, confusion, sleepy tendency at daytime, or muscle weakness, and providing education on precautions for fall risk and how to use the nurse-call bell. The care plans were mapped onto the local phrases at each site. Phrases that had not previously been mapped locally were added to the local nursing statements.

The CDS systems were disseminated and implemented at each site with different schedules according to their logistical and administrative situations. One hospital had implemented and applied the CDS to all inpatient units 5 years previously,^40^ another had done this 2 years previously, and the remaining 2 were currently implementing it.

## MATERIALS AND METHODS

### Study sites and retrospective modeling-data profile

The participating institutions were 3 tertiary academic hospitals and 1 public hospital having >800 beds in the metropolitan area of Seoul, South Korea. Three of them used self-developed EMR systems, and the fourth had used a commercial EMR system for >15 years. For electronic nursing records, 1 hospital adopted the nursing statements approach using the ICNP, 2 used North American Nursing Diagnosis Association (NANDA) classification, and the other hospital used NANDA, Nursing Interventions Classification (NIC), and ICNP (Table 1).

**Table 1.** Retrospective modeling-data profile of the participating hospitals

	Site A	Site B	Site C	Site D
SNTs used in EMR systems	ICNP version 1.0	NANDA	NANDA	NANDA, NIC, and ICNP version 1.0
No. of units	6	6	9	6
Data retrieval period	1 year	2 years	1.5 years	4 years
	(September 2014 to August 2015)	(June 2014 to May 2016)	(January 2017 to June 2018)	(July 2015 to June 2019)
No. of admissions	14 307	21 172	31 930	36 314
Hospital days	122 179	172 592	294 268	296 013
No. of falls	220	292	357	525
Fall rate per 1000 hospital-days	1.95	1.69	1.25	1.77
Fall-related injury rate per 1000 hospital-days	0.44	0.40	No data	0.54

NANDA: North American Nursing Diagnosis Association; NIC: Nursing Interventions Classification; ICNP: International Classification for Nursing Practice; SNTs: standardized nursing terminologies; EMR: electronic medical record.

The data-retrieval periods were set at 1–4 years according to the estimated average fall rates. The fall rates recorded in the data were 1.25–1.95 per 1000 hospital-days, and number of fall events ranged from 220 to 525. Local cohort data were all preprocessed according to the inclusion criteria of aged ≥18 years and admitted for at least 24 h, and the exclusion criterion of having a psychiatric, obstetric, emergency, or pediatric condition. The synthetic minority oversampling technique was applied to nominal and continuous features to address the data imbalance between events and nonevents. Each cohort was split at a 7:3 ratio into the training and validating. This study was reviewed and approved by the IRBs of the participating hospitals, and the requirement to obtained patient consents was waived.

### Comparison of nursing statements in hospitals

The coded nursing statements at the sites were compared semantically using The Catalog, with the local mapping process conducted by the research team at each site. Two of the 4 hospitals already had mapping experience from the previous eMeasurement population study. We followed the process that involved internal and external nursing informaticians from The Catalog study. These mapping results were aligned by assessment, diagnosis, and outcome (7 care elements: physiological, therapeutics, cognitive factors, risk behaviors, communication ability, situational factor, and physical environment), and intervention (7 care elements: universal care, environmental management, risk-targeted care, protocol provision, education provision, information sharing, and alarm monitoring). The protocol provision care element was not explicitly expressed in statements at most sites, so we combined it with the risk-targeted care element.

### Comparison of the 4 implementation models

The prediction concept model consisted of 5 constructs: patient demographics and administrative information, medication, nursing interventions, nursing assessment and diagnosis, and fall-risk assessment tools.^24^ Two hospitals used the Morse Fall Scale as a fall-risk assessment tool, while the other 2 used the Hendrich II Fall Risk Model and STRATIFY.

The Korean Patient Classification System (KPCS) was included in prediction models as an administrative information feature set. This is a standardized system developed by the Korean Hospital Nurses Association that classifies patients into 6 groups based on their nursing needs, from group 1 (fewest needs) to group 6 (most needs), and is used to estimate nursing staff needs.^41^ We used 7 of the 50 items in the KPCS: exercise (transfer, ambulation status), treatment (tube management), medication (intravenous fluid exchange), surveillance (consciousness/orientation, circulation/sensory/movement), education (communication problems), and total score. KPCS data were unavailable at site D.

We used the following evaluation metrics to compare the 4 prediction models: sensitivity, specificity, positive predictive value, negative predictive value, and receiver operating characteristics (ROC) analysis. Sensitivity tests were used to assess how the care plan constructs contributed to the fall-risk predictions of each implementation model by examining decreases in variation. The KPCS features were separated from the administrative construct in the sensitivity tests. The fall-risk assessment-tools construct was excluded from the models implemented using the CDS systems due to a request from the hospitals to decrease the burden on their nurses. Most items of the heuristic tools were redundant with other model features (eg, KPCS) or nursing assessments. For example, previous fall history was a strong risk factor included in most heuristic tools, and this variable was replaced with the data element of initial nursing history as determined at admission.

### Pattern of longitudinal patient outcomes and nursing activities

**Figure 2.**
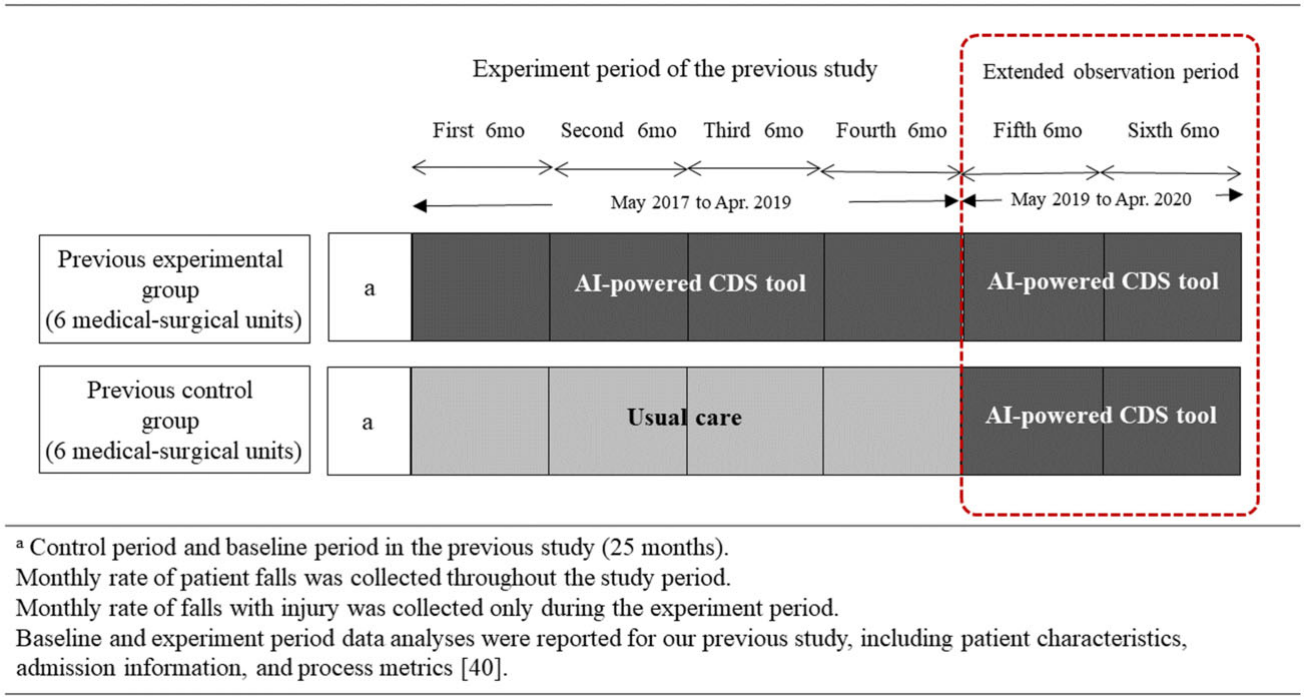
Extended observation period and data collection points based on the previous study design for longitudinal data analysis in this study.

We selected the hospital site that had used the CDS for longest (since April 2017) to observe longitudinal outcome patterns. We previously performed an experimental study of a controlled interrupted time series at that site.^40^ After that quasi-experimental study, the hospital deployed the CDS tool to all 24 units, including 6 constituting a control group in the previous study. We were able to observe the longitudinal changes in the 2 groups from enterprise deployment until the hospital was seriously affected by the COVID-19 pandemic in 2020 (Figure 2). The slope patterns of patient outcomes (ie, fall rates) were evaluated using interrupted time-series analysis (ITSA). The fall rate was calculated as the total number of falls per 1000 hospital-days each month. The patient outcomes in this study were determined using both self-reports from the quality assurance department at each site and chart reviews that were conducted by our research team using a rule-based algorithm to detect the typical expressions used to describe falling in nursing notes and eye screening. For unclear cases, the local research teams conducted full chart reviews and made final judgments. We observed the relationships between the outcomes and nursing activities. The average frequency of nursing activities provided to patients was also evaluated over time. We used Stata software (version 15.1, StataCorp, TX, USA) to perform the statistical analyses.

## RESULTS

### Comparison of the local nursing statements in hospitals

Each hospital covered each care element differently using The Catalog (Table 2). Sites C and D had greater coverage than sites A and B. Among the assessment, diagnosis, and outcome statements, the therapeutics, behavior risks, situational factors (eg, residing caregiver/bedside sitter), and physical environment care elements were insufficient at all 4 hospitals. Regarding the intervention statements, there were fewer than 50% of the care elements of information sharing and alarm monitoring at 3 of the 4 hospitals.

**Table 2.** Comparison of coverage of the standard statements in the ICNP-based inpatient fall-prevention catalog (‘The Catalog’) among the hospitals

			No. of mapped local statements (%)			
Care elements in The Catalog		No. of statements	Site A	Site B	Site C	Site D
Assessment, diagnosis, and outcome	Pathophysiological	19	19 (100%)	10 (52.6%)	18 (94.7%)	19 (100%)
	Therapeutics	7	0	1 (14.3%)	6 (85.7%)	2 (28.6%)
	Cognitive factors	10	4 (40.0%)	5 (50.0%1)	5 (50.0%)	10 (100%)
	Risk behaviors	3	0	1 (33.3%)	1 (33.3%)	1 (33.3%)
	Communication ability	1	1 (100%)	1 (100%)	1 (100%)	1 (100%)
	Situational factors	1	1 (100%)	0	0	0
	Physical environment	1	0	0	0	0
Intervention	Universal care	13	10 (76.9%)	5 (38.5%)	13 (100%)	10 (76.9%)
	Environmental management	11	3 (27.3%)	9 (81.8%)	3 (27.3%)	11 (100%)
	Risk-targeted care and protocol provision	19	7 (36.8%)	10 (52.6%)	10 (52.6%)	19 (100%)
	Education provision	6	6 (100%)	4 (66.7%)	6 (100%)	6 (100%)
	Information sharing	5	1 (20%)	1 (20%)	2 (40.0%)	5 (100%)
	Alarm monitoring	2	0	0	0	1 (50.0%)
Total		98	52 (53.1%)	47 (48.0%)	65 (66.3%)	85 (86.7%)

### Comparison of the implementation models at the 4 sites

Four of the 6 constructs in the concept model were successfully mapped to local data elements. The fall-risk assessment-tools element was mapped exactly to the corresponding data elements, with a few differences across the tools. For example, the Hendrich II Fall Risk Model and STRATIFY tool did not include the concepts of previous fall history and medication, respectively. The concepts of demographics and administrative information were mapped optimally, while medication concepts were mapped to local drug classifications due to the lack of common classifications.

The evaluation metrics indicated that the prediction performance of the models was acceptable whether or not the heuristic tools construct was used (Table 3). The areas under the ROC curves (AUROCs) of the optimized implementation models were 0.9309–0.9851, indicating good performance. After excluding the heuristic tools construct, the AUROCs of the implementation models were 0.8051–0.9581. For both of these the performance was better than for the heuristic tools alone (AUROC = 0.6491–0.7364).

**Table 3.** Evaluation metrics of implementation models

	Model with the heuristic tools construct **Model without the heuristic tools construct**			
	Site A	Site B	Site C	Site D
Sensitivity	0.9490	0.9521	0.8634	0.9359
	**0.6544**	**0.6609**	**0.8584**	**0.9089**
Specificity	0.8262	0.9055	0.8751	0.8563
	**0.7812**	**0.8939**	**0.8812**	**0.8871**
Positive predictive value	0.8445	0.8145	0.4240	0.3953
	**0.4012**	**0.4426**	**0.4348**	**0.4376**
Negative predictive value	0.9422	0.9775	0.9837	0.9925
	**0.9255**	**0.9539**	**0.9832**	**0.9902**
AUROC	0.9573	0.9851	0.9309	0.9643
	**0.8051**	**0.8740**	**0.9334**	**0.9581**

AUROC: area under the receiver operating characteristics curve.

**Figure 3.**
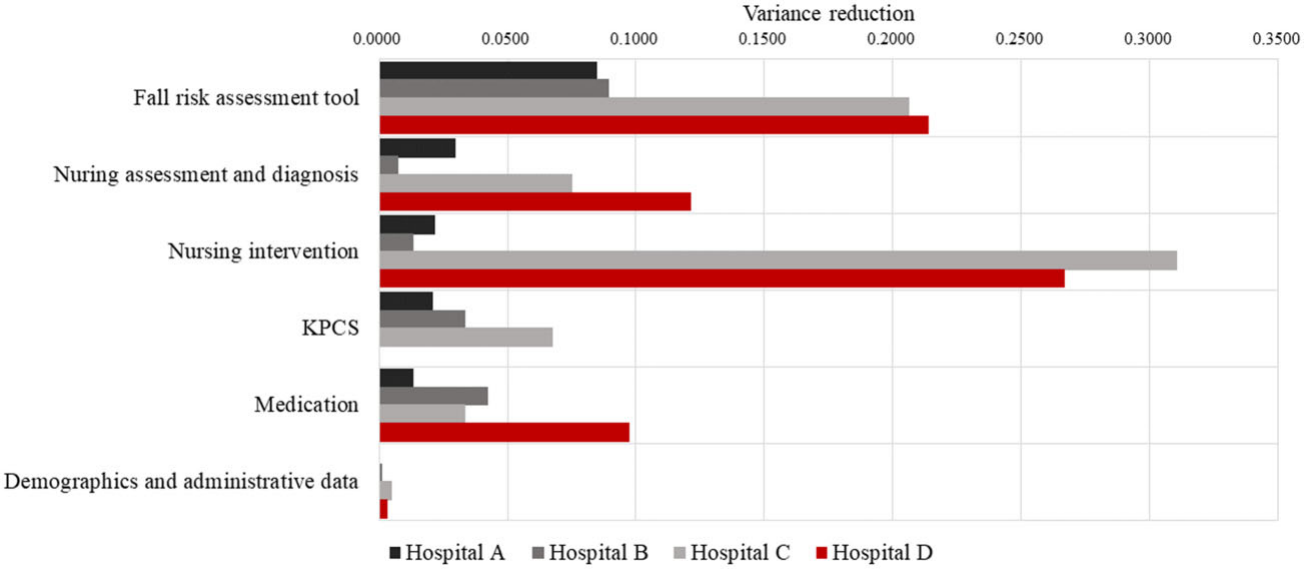
Comparison of the variance reductions of the 4 implementation models for fall-risk predictions. KPCS: Korean Patient Classification System.

Sensitivity tests of the contribution of each construct were synchronously varied among the 4 sites (Figure 3). The heuristic tools category generally contributed greatly to fall predictions. Specifically, the Morse Fall Scale at Sites C and D contributed more than the Hendrich II Fall Risk Model and STRATIFY did at Sites A and B. Two nursing process categories (assessment and diagnosis, and intervention) differed the most among the hospitals. KPCS, medication, and demographic and administrative information made relatively small contributions.

### Pattern of longitudinal patient outcomes and nursing activities

**Figure 4.**
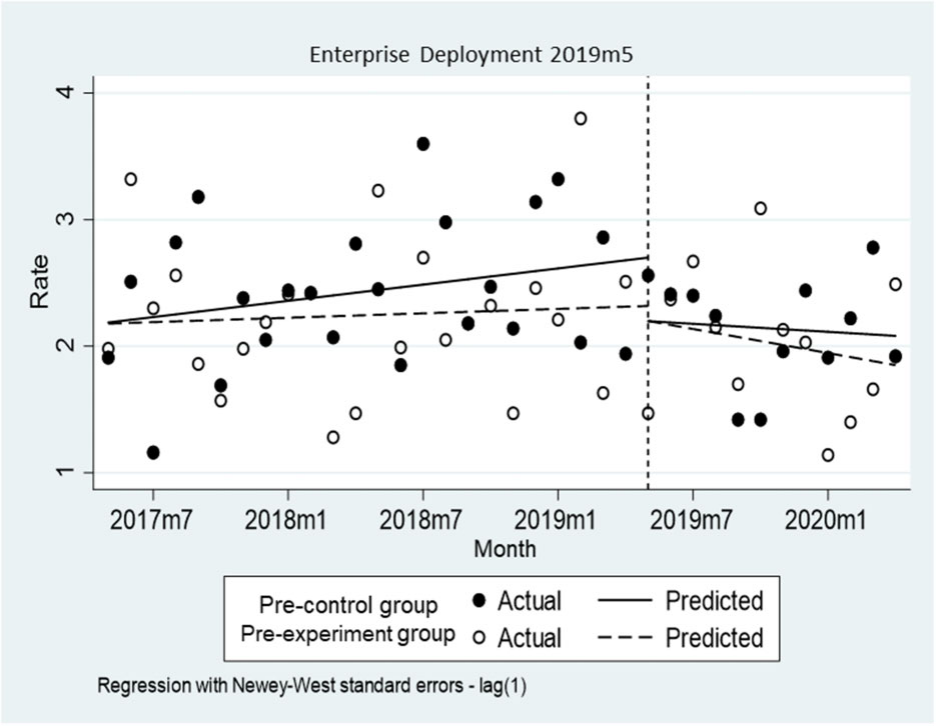
Multigroup interrupted time-series analysis with Newey-West standard errors and one lag.

The fall rates per 1000 hospital-days for the control group in the previous study increased monotonically by 0.21 prior to May 2019 (*P *=* *.158, CI = −0.01 to 0.05) (Figure 4). In the first month after CDS tool deployment, a −0.50 decrease was observed in the fall rate (*P *=* *.160, CI = −1.21 to 0.21), followed by a − 0.03 decrease in the monthly trend (*P *=* *.354, CI = −0.10 to 0.04). That of the experimental group in the previous study was also observed to increase slightly by 0.01 prior to May 2019 (*P *=* *.722, CI = −0.03 to 0.04). A − 0.12 decrease was observed in the fall rate (*P *=* *.758, CI = −0.89 to 0.66) in June 2019, followed by a − 0.04 decrease (*P *=* *.458, CI = −0.14 to 0.06). The initial mean level difference between the 2 groups was not significant in the multiple-group ITSA (*P *=* *.976, CI = −0.59 to 0.61), nor was the difference in the mean baseline slope (*P *=* *.491, CI = −0.03 to 0.06).

**Figure 5.**
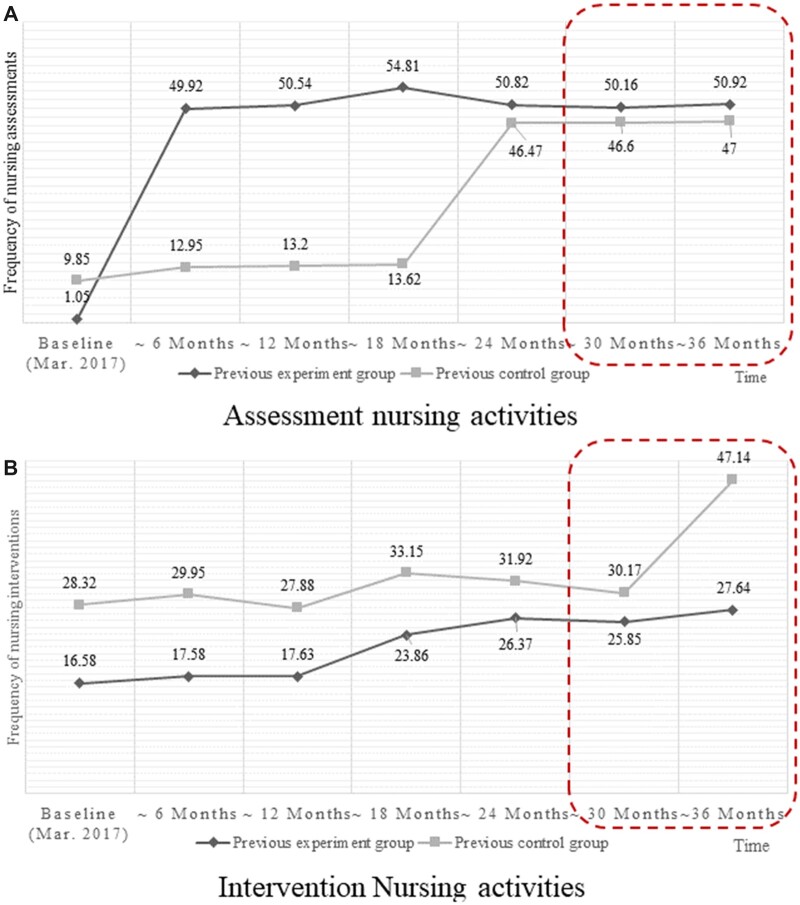
Changes in patterns of nursing activities in the 2 groups. Dotted boxes indicate the period after the enterprise deployment of the CDS tool.

The assessment nursing activity was maintained continuously after May 2019 (Figure 5). Nursing intervention activities increased notably in the previous control group but only slightly in the experimental group in the previous study.

## DISCUSSION

This study used 4 hospitals and the local nursing terms and statements in their EMR systems to determine if standard terminologies can ensure the consistent development and deployment of an AI-powered CDS tool. Our postimplementation approach of using SNTs was feasible and practical. The 4 hospitals successfully implemented optimized models, which exhibited acceptable performance. The CDS tools including the prediction models will help nurses in their practical work. The coverage of local nursing statements varied among the hospitals. The local nursing terms and statements of each hospital were inadequate for expressing the fall-prevention care elements of nursing diagnosis, assessment, outcome, and intervention. This suggest that practices varied between the sites or that nurses were unable to adequate express their care activities due to weaknesses of their statements. The sensitivity tests for the optimized models revealed different contributions to fall-risk predictions, which is related to synchronous variations in machine learning. The longitudinal tracking of patient outcomes at one site revealed no significant changes over time in outcomes. However, we observed positive patterns in nurse behaviors and patient outcomes, which could require the diachronic evolution of the prediction models.

The necessity for SNTs in clinical information systems is clear, since they describe nursing practices and concepts in the systems, express nursing knowledge consistently, and can communicate at multiple levels. They are also required to aggregate and analyze data for purposes such as quality improvement, research, reimbursement, and policy development.^1^^,^^42^^,^^43^ They should have sufficient domain coverage and interoperability among computable nursing terminology systems. The use of SNTs is aimed at improving the consistency, content, and format of nursing communications to enhance the effectiveness and efficiency of information sharing among nurses and the public.^2^^,^^44^^,^^45^ All hospitals need to adopt SNTs when implementing EMR systems to obtain these potential benefits, but many South Korean hospitals adopted EMRs during 2000–2010 using either self-developed or purchased commercial products, and the South Korean government has avoided endorsing nationwide SNTs until 2015.^46^ SNTs were therefore not adopted in commercial products, and self-developed EMR systems also did not comprehensively accept SNTs.

The adoption of SNTs in EMRs was insufficient to link practice activities with patient outcomes. Relationships among nursing-process components represented by SNTs are essential to achieve the potential of SNTs and improving nursing knowledge.^47^ From an EMR system standpoint, practice activities of nurses are more specific than those expressed by SNTs, such as assessing and measuring patients, and collecting, interpreting, and documenting clinical data. These clinical data elements should also be linked to SNTs. These relationships, which have often been missed in current EMR systems, were a revision recommendation made by the reviewers of ISO 18104:2003 (the standard for categorical structures for the representation of nursing diagnoses and nursing actions in terminological systems).^1^

Gomes et al^37^ suggested using the “reason for the request” and “clinical indication for requesting an intervention” in the openEHR archetypes to link nursing interventions with diagnoses. However, this method does not explicitly determine nursing interventions. The 3N linkage, which refers to linking the NANDA-International (NANDA-I), NIC, and NOC, has been used the most for SNTs in practice, but its use is still not common.^48^ Zhang et al^48^ highlighted the accuracy problem of nursing diagnoses based on identifying signs, symptoms, and diagnostic etiology. A recent study^49^ explored the nursing diagnoses used in 4 tertiary and academic hospitals that claimed to adopt NANDA-I in their EMR systems, and found that only 40% of nursing diagnoses were mapped across hospitals and only 65 terms among them were derived from the NANDA-I. This means that more than half of the nursing diagnoses used in practice were local ones, rather than being based on SNTs. The Catalog approach was recommended by the ICN,^45^ and we consider it a useful content model, and that nursing-sensitive outcome domains should be the highest-priority areas for an approach. The Catalog guided all postimplementation processes and enabled consistent data collection, comparison, and analysis among the 4 hospitals.

We optimized the concept model for each site and found around 15% synchronic variation among the models. Synchronic variation refers to the differences that emerge between copies of an adaptive machine-learning system implemented at different sites or among different patients in machine learning.^50^ In this study, the models implemented at the 4 sites in this study were not copies; instead, they stemmed from the same concept model, but the final feature sets became slightly different depending on the availability of data in each EMR and the data sets from which the algorithm learns. For example, the model for site D did not include the KPCS features. For high-risk medications, the model for site A only had 4 drug classes, while that at site B had 11. The other 2 models/sites had 7 and 8 drug classes. These variations arose from differences in patient population, local policies, organizational culture, and user behaviors.

The users in our previous study reported positive attitudes toward AI-powered CDS tools.^51^ Two other previous real-world effectiveness studies applied an AI-powered CDS tool to other hospitals,^40^^,^^52^ and found that it had enough potential for decreasing fall incidents and fall-related injuries as well as for the real-time longitudinal tracking of nursing activities. Analysis of data warehouses indicated that the tasks were previously time-consuming and physically intense. The Korea Institute for Healthcare Accreditation recognized the usefulness of the AI-powered CDS tool for fall prevention in their program for healthcare organization accreditation.

While no significant longitudinal fall-rate trend was found in this study, increased nursing assessment activity and changes in nurse behavior were observed. These diachronic behavior changes suggest a sign for improvements in patient outcomes,^53^ and also require the prediction model to be updated.^50^ Recent trends in healthcare information technology provide new opportunities for CDS, and an adaptive CDS system is recommended over a static one. Adaptive CDS systems can change their performance over time via learning, and interpret data using new clinical evidence, data types and resources, and methods.^10^ We found changes in user responses to the CDS tool through longitudinal observation, which might change the performance of the prediction model. To update an adaptive CDS, a data pipeline should be installed for support, and SNTs and clinical content would be important components.

This study had 2 main limitations. Frist, despite using a well-established previously defined mapping protocol and the mappings being conducted by local research teams, the statistical reliability of the mapping accuracy was not measured. Second, no statistical changes in patient fall-rate trends were observed during the 6-month follow-up period, which might have been too short to detect any significant changes in patient outcomes. We need to further follow-up the effects of the AI-powered CDS tool in a study with a multisite approach.

An important strength of this study was addressing the possibility of representing evidence-based nursing knowledge consistently in the form of an AI-powered CDS tool using SNTs across different settings. Achieving this would allow patient outcomes and nursing indicators to be aggregated in the standardized format using the SNTs among multiple sites. This study approach provided the opportunity to further utilize SNTs in AI-powered CDS tools for other hospital-acquired conditions and nursing-sensitive outcomes.

Our research team is currently attempting to apply copies of implementation models to other secondary hospital settings where structured data are rare and most nursing records are documented as free text. It is difficult to aggregate local training data sets and establish SNT use in these settings.

## CONCLUSIONS

SNTs including LOINC and ICNP demonstrated semantic interoperability in deploying an AI-powered fall-prevention CDS tool across 4 hospitals with different local terms and nursing statements in their EMR systems. The ICNP-based fall prevention tool The Catalog, which was developed based on a patient safety framework and clinical guidelines, acted as clinical content to consistently and meaningfully guide the mapping of local nursing assessments, diagnoses, outcomes, and interventions. The multisite application of the fall-risk prediction model revealed few notable synchronic variations among the sites. Even short-term follow-up surveillance of how the model performs and its effects on patient outcomes can reveal meaningful changes in nursing practice patterns. These systematic approaches, leveraged by the SNTs and clinical content, suggest opportunities for regional-, national-, and international-level nursing data analyses and comparisons.

## Supplementary Material

ocad145_Supplementary_Data

## Data Availability

The data that support the findings of this study are available from the authors upon reasonable request and with permission of the study hospitals.
