# Supplementary material for: Utilizing standardized nursing terminologies in implementing an AI-powered fall-prevention tool to improve patient outcomes: a multihospital study
Source: J Am Med Inform Assoc. 2023 Jul 28;30(11):1826–36. doi: 10.1093/jamia/ocad145 (PMC10586045; doi:10.1093/jamia/ocad145)
Supplement: ocad145_Supplementary_Data [file ocad145_supplementary_data.docx]

**Supplement Materials**

Table 1. Representation of a fall prediction model’s constructs with standard nursing terminology

| Model construct | | Group of concepts | Concept | | Mapping result of SNT  (ICNP/LOINC code) | |
| --- | --- | --- | --- | --- | --- | --- |
| Demographics and administrative information | | Demographics | Age | | Age calculated (29553-5) | |
|  |  |  | Sex | | Sex [HL7.v3] (72143-1) | |
|  |  | Administrative information | Primary medical diagnosis | | Primary diagnosis (18630-4) | |
|  |  |  | Medical department | | Hospital department (284548004)^*^ | |
|  |  |  | Nursing unit | | Care setting Facility [NHCS](78022-1) | |
|  |  |  | Days of stay | | hospital stay duration(78033-8) | |
|  |  |  | Secondary diagnoses | | Secondary diagnosis (81885-6) | |
|  |  | Korean patient classification system | Exercise: Transfer | | Ability to transfer (10000204) | |
|  |  |  | Exercise: Ambulation status | | Ability to mobilize (10012108) | |
|  |  |  | Treatment: Tube management | | Tube (10020216), managing (10011625) | |
|  |  |  | Medication: IV exchange | | Managing Fluid therapy (10042096) | |
|  |  |  | Surveillance: Consciousness/orientation | | Assessing consciousness (10050186), Assessing orientation (10043752) | |
|  |  |  | Surveillance: circulation/sensory/movement | | Assessing circulatory system (10050193), Assessing body movement (10050223 ), Sensory ability (10024035) | |
|  |  |  | Communication problem | | Identifying Obstruction To Communication (10009683) | |
| Medications | | Medications increasing fall-risk | Antidepressant^*^ | | N06A, N06AX, N06CA | |
|  |  |  | Anxiolytics^*^ | | N05B, N05BX | |
|  |  |  | Antiemetic^*^ | | A04, A04A, A04AD | |
|  |  |  | Sedatives^*^ | | N05C, N05CM, N05CX | |
|  |  |  | Antiepileptic^*^ | | N03, N03A, N03AX | |
|  |  |  | Diuretics^*^ | | C02* ~C03*, C07*~C09* | |
|  |  |  | Analgesics^*^ | | N02, N03, A03*, C03* | |
|  |  |  | Antihypertensive: vasodilator^*^ | | C01D, C01DB(X), C07E(A,B) | |
|  |  |  | Antiarrhythmic^*^ | | C01B, C01B* | |
|  |  |  | Antihistamine^*^ | | D06, R06 | |
|  |  |  | NSAIDs^*^ | | M01A, M01AX, S01BC | |
| Fall-risk assessment tool | |  | Morse Fall Scale | | Assessment tool (10002832), assessing risk of falls (10023520) | |
|  |  |  | STRATIFY | |  |  |
|  |  |  | Hendrich II | |  |  |
| Nursing diagnosis and assessment | | Pathophysiologic factors | Risk of fall injury | | Risk for fall-related injury (10038521) | |
|  |  |  | Bleeding tendency | | Bleeding (10003303), risk (10015007) | |
|  |  |  | Risk of bone disease (e.g. arthritis, total hip replacement, knee replacement history) | | Bone health^†^ | |
|  |  |  | Visual impairment | | Impaired vision (10022748) | |
|  |  |  | Hearing impairment | | Impaired hearing (10022544) | |
|  |  |  | Balancing impairment (related to cerebellum, vestibular organ) | | Impaired balance (10047170) | |
|  |  |  | Gait impairment | | Impaired walking (10001046) | |
|  |  |  | Physical mobility impairment – paralysis | | Paralysis (10022674) | |
|  |  |  | Motor weakness | | Weakness (10024897), Fine motor function (10043026) | |
|  |  |  | General weakness | | Weakness (10022880) | |
|  |  |  | Physical mobility impairment - musculoskeletal problem | | Abnormal (10013269), Musculoskeletal status (10034292) | |
|  |  |  | Use of ambulatory aids | | Walking using device (10020903) | |
|  |  |  | Urinary elimination alteration | | Impaired urination (10021790) | |
|  |  |  | Incontinence | | Bowel incontinence (10027718), Urinary incontinence (10026895) | |
|  |  |  | Frequent toilet access | | Frequent toileting^†^ | |
|  |  |  | Dizziness | | Dizziness (10045584) | |
|  |  |  | Vertigo | | Vertigo^†^ | |
|  |  |  | Syncope experience during hospitalization | | Syncope^†^, History^†^ | |
|  |  |  | Orthostatic hypotension in the last 7 days | | Hypotension (10009534), History^†^ | |
|  |  | Therapeutics | Pain | | Assessing pain (10026119) | |
|  |  |  | Medications increasing fall-risk | | Risk for fall (10015122), Medication (10011866) | |
|  |  |  | History of adverse drug reactions | | Adverse medication interaction (10042716), History^†^ | |
|  |  |  | Use of catheter(s) or line(s) | | Catheter (10004087) | |
|  |  |  | Use of drain tube(s) | | Drainage tube (10046109) | |
|  |  |  | Use of an indwelling Foley catheter | | Urinary catheter (10020373) | |
|  |  |  | Restraint use | | Restraint (10017164) | |
|  |  | Mental, Cognitive factors | Decreased cognitive function/status | | Cognition (10004485) | |
|  |  |  | Dementia | | Dementia (10031091) | |
|  |  |  | Delirium | | Delirium (10005692) | |
|  |  |  | Disorientation | | Disorientation (10001235) | |
|  |  |  | Emotional state of excitement | | Excitement^†^ | |
|  |  |  | Agitation | | Agitation (10002035) | |
|  |  |  | Decreased consciousness level | | Consciousness (10004975) | |
|  |  |  | Expression of anxiety | | Anxiety (10000477) | |
|  |  |  | Fear of falling | | Fear (10000703). Fall (10029405) | |
|  |  |  | Uncooperative status for clinical staff | | Uncooperative behavior^†^ | |
|  |  |  | Noncompliance with safety precaution | | Non adherence (10001371) | |
|  |  | Behavior risk | History of falling | | Fall (10029405), History^†^ | |
|  |  |  | Sleep pattern disturbance | | Impaired sleep (10027226) | |
|  |  |  | History of long-term fall-risk drug medication | | Medication (10011866), History^†^ | |
|  |  | Communication ability | Impaired verbal communication | | Ability to communicate (10000052) | |
|  |  | Situational factor | Caregiver/bed-side sitter reside | | Family caregiver (10007565), Bedside sitting^†^ | |
|  |  | Physical environment | Environmental risk factors in the room | | Environmental safety (10031247) | |
| Model construct: Nursing intervention | | | | | |  |
| Group of concepts | Term or Statement of The Catalog | | | Concept mapping result  (ICNP® code) | |  |
| Universal care | Provides fall prevention protocol with risk assessment at admission | | | Assessing risk for falls on admission (10037435) | |  |
|  | Demonstrate how to use a nurse call bell from the patients' bedside | | | Demonstrating (10005713), Call system device (10003825) | |  |
|  | Keep frequently used objects within easy reach from patients' bed  (utensil/ call bell/ mobile phone/ remote control/ water bottle/ etc.) | | | Arranging (10002527), Patient's belongings^†^ | |  |
|  | Use of side rails and educate/explain to the patient and family that the main purpose of the side rails is to assist the patient to move and turn in the bed (not to cross over the side rails) | | | Putting on OR in (10016201), Bed rail (10003201) | |  |
|  | keep beds in a low position | | | Lowering (10011455), Bed (10003168) | |  |
|  | Lock the bed wheels | | | Locking^†^, Bed wheel^†^ | |  |
|  | Educate/explain/demonstrate to lock the brakes before getting out or into the wheelchair | | | Locking^†^, Wheelchair wheel^†^ | |  |
|  | Keep the floor dry | | | Maintaining (10011504), (room) Floor safety^†^ | |  |
|  | Check the lighting condition so that it does not interfere with the behavior or movement in the room | | | Assessing (10002673), Room light^†^ | |  |
|  | Organize beds and arrange personal care items | | | Arranging (10002527), Bedside^†^ | |  |
|  | Conduct purposeful nursing rounds at regular intervals (room environment, bedding, organizing, lighting, floor, poles, call bell, identify patient needs) | | | Performing (10014291), (regular) Rounding^†^ | |  |
|  | (When going to the toilet / changing position / moving) Be sure to call a nurse | | | Providing (10015935), Call system device (10003825) | |  |
|  | Notify the next nursing rounding/visit time | | | Providing (10015935), (regular) Rounding^†^, Schedule^†^ | |  |
| Environmental management | Conduct regular environmental management rounding | | | Performing (10014291), Environmental safety (10031247), (regular) Rounding^†^ | |  |
|  | Periodically check the walking aids | | | Checking device safety (10030924) | |  |
|  | Install a convex mirror in the hallway. | | | Installing (10010353), Skewed mirror^†^ | |  |
|  | Install falls precaution signs | | | Installing (10010353), Precaution sign/visual indicator^†^ | |  |
|  | Ensure that the floor is not slippery. | | | Maintaining (10011504), Clean/ Dry floor^†^ | |  |
|  | Minimize general noise at room | | | Decreasing noise (10050384) | |  |
|  | Perform cleaning at times of low walking | | | Adjusting (10001760), Room cleaning^†^ | |  |
|  | Keep assistant nursing personnel/ caregiver/ bed-side sitter with patient. | | | Keeping family caregiver (10007565), Bedside^†^ | |  |
|  | Raise the bed railing, using the railing cover | | | Putting on OR in (10016201), Bedrail (10003201) | |  |
|  | Remove sharp edges of furniture | | | Checking furniture safety^†^ | |  |
|  | Lower or remove the threshold | | | Checking room floor safety^†^ | |  |
| Risk-targeted care | Assess and monitor fall risk and risk factors consistently (using validated tools or methods) | | | Assessing risk for falls (10023520) | |  |
|  | Provide cognitive orientation regularly | | | Providing (10015935), Orientation (10013810) | |  |
|  | Regularly assist to toilet access | | | Assisting with toileting (10023531) | |  |
|  | Be sure to call the nurse when moving out / on the bed. | | | Providing (10015935), Call system device (10003825) | |  |
|  | Provide precaution on high risk of falls related to medication | | | Teaching patient (10033126), Call system device (10003825) | |  |
|  | Determine the number of personal assistants during transportation | | | Checking (10004189), Number of assistants^†^ | |  |
|  | Attention to night movements and behaviors due to sleep disturbances | | | Teaching patient (10033126), Risk for fall (10015122), (related to) Impaired sleep (10012929) | |  |
|  | Re-assess/monitor pain | | | Assessing pain (10026119) | |  |
|  | Encourage a use of aid devices to prevent falls | | | Promoting walking using device (10037636) | |  |
|  | Provides portable toilet (e.g. Bed pan, Comodo) | | | Providing (10015935), Bed pan (10003199 )/comodo^†^ | |  |
| Provision of protocol | Apply restraint protocol | | | Providing (10015935), Restraint (10017164), (with) Protocol (10015926) | |  |
|  | Provide a toilet scheduling | | | Toilet schedule^†^ | |  |
|  | Assign the room to close to the nursing station and apply frequent rounding | | | Adjusting (10001760), Room assignment^†^ | |  |
|  | Request physical / occupational therapy for rehabilitation department | | | Referring to physical therapy (10024019) | |  |
|  | Provide safe transfer and transportation of the patient in the standard protocol | | | Assisting (10002850), Movement (10012274), (with) Protocol (10015926) | |  |
|  | To minimize the problem of cognitive impairment, maintain consistency of work schedule such as visiting schedule, procedure, assignment of charge | | | Maintaining (10011504), Consistency^†^, Usual tasks^†^ | |  |
|  | Control the environment and interventions to identify and minimize the drivers of impulsive behavior. | | | Environmental safety management (10042507) | |  |
|  | Check the stability of the table, chair handle, furniture, etc. | | | Checking (10004189), Environmental safety (10032147) | |  |
|  | Wear seat belt when walking/ moving | | | Applying safety device (10002472) | |  |
| Provision of education | Educate patients and their families for prevention of falls | | | Teaching family about fall prevention (10040269) | |  |
|  | Conduct fall prevention education to family members/ caregivers/ bed-side sitters. | | |  |  |  |
|  | Educate patients for prevention of falls | | | Teaching about fall prevention (10040253) | |  |
|  | Demonstrate the use of pedestrian devices and train usage | | | Educating (10006564), Use of walking device^†^ | |  |
|  | Encourage early ambulation and regular exercise | | | Encouraging (10006823), Exercise behavior (10007294) | |  |
|  | Make sure standing up slowly when a patient is over bed. | | | Encouraging (10006823), Ambulation technique (1000222) | |  |
| Information sharing | For patients at high risk for fall, open communication, shared responsibilities for planning with the next shift and other team | | | Communicating^†^, Risk for fall (10015122) | |  |
|  | Share fall risk information with patients using visual fall markers | | | Communicating^†^, Risk for fall (10015122), Visual indicator^†^ | |  |
|  | Keep caregivers/bed-side sitter on bed due to at-risk for falls | | | Arranging (10002527), Caretaking by caregiver (10035388) | |  |
|  | Share the information for patients at high risk for fall with doctors and other healthcare providers | | | Collaborating with interprofessional team (10039416), Physician (10014522) | |  |
|  | Share the information about the fall risk factors with the guardian/caregiver for fall prevention, and engage patients and family | | | Communicating risk for fall (10015122), Caregiver (10003958) | |  |
| Alarm monitoring | Adopt the medical alarm system for fall prevention upon agreement of a patient/guardian | | | Installing (10010353), Fall safety alarm (10041518) | |  |
|  | Monitor the medical alarm system for fall prevention | | | Maintaining fall safety alarm (10041525) | |  |

^†^ indicates terms not mapped to ICNP; * means drug classes mapped to WHO’s ATC code

Figure 1. Mapping relationships between the prediction model’s constructs and concepts represented by SNTs, and local EMR data elements and nursing statements
